# TRIM5α Modulates Immunodeficiency Virus Control in Rhesus Monkeys

**DOI:** 10.1371/journal.ppat.1000738

**Published:** 2010-01-22

**Authors:** So-Yon Lim, Thomas Rogers, Tiffany Chan, James B. Whitney, Jonghwa Kim, Joseph Sodroski, Norman L. Letvin

**Affiliations:** 1 Beth Israel Deaconess Medical Center, Harvard Medical School, Boston, Massachusetts, United States of America; 2 Department of Cancer Immunology and AIDS, Dana-Farber Cancer Institute, Harvard Medical School, Boston, Massachusetts, United States of America; King's College London School of Medicine, United Kingdom

## Abstract

The cytoplasmic TRIM5α proteins of certain mammalian lineages efficiently recognize the incoming capsids of particular retroviruses and potently restrict infection in a species-specific manner. Successful retroviruses have evolved capsids that are less efficiently recognized by the TRIM5α proteins of the natural hosts. To address whether TRIM5α contributes to the outcome of retroviral infection in a susceptible host species, we investigated the impact of *TRIM5* polymorphisms in rhesus monkeys on the course of a simian immunodeficiency virus (SIV) infection. Full-length TRIM5α cDNAs were derived from each of 79 outbred monkeys and sequenced. Associations were explored between the expression of particular *TRIM5* alleles and both the permissiveness of cells to SIV infection *in vitro* and clinical sequelae of SIV infection *in vivo*. Natural variation in the TRIM5α B30.2(SPRY) domain influenced the efficiency of SIVmac capsid binding and the *in vitro* susceptibility of cells from the monkeys to SIVmac infection. We also show the importance *in vivo* of the interaction of SIVmac with different allelic forms of TRIM5, demonstrating that particular alleles are associated with as much as 1.3 median log difference in set-point viral loads in SIVmac-infected rhesus monkeys. Moreover, these allelic forms of *TRIM5* were associated with the extent of loss of central memory (CM) CD4+ T cells and the rate of progression to AIDS in the infected monkeys. These findings demonstrate a central role for TRIM5α in limiting the replication of an immunodeficiency virus infection in a primate host.

## Author Summary

The cytoplasmic TRIM5α restricts the replication of a broad range of retroviruses in a species-specific manner. In the present study we show that TRIM5α is more than a species barrier for retroviruses. We show that naturally occurring B30.2(SPRY) polymorphisms affect retrovirus infection. These observations demonstrate the importance of SIV/B30.2(SPRY) interactions *in vivo*. These findings are the first demonstration of the importance of such a pathogen/host protein interaction *in vivo*. Importantly, the striking variability in the clinical course of HIV-infected individuals has long puzzled the biomedical community. A large number of investigators have devoted considerable effort to determine what genetically determined factors might contribute to the containment of HIV replication, reasoning that an understanding of the determinants of effective control of HIV spread will provide important targets for both drug and vaccine development. Our demonstration in the present study that B30.2(SPRY) polymorphisms have a dramatic effect on the clinical outcome of an AIDS virus infection highlight the extraordinary importance of TRIM5α on the control of an AIDS virus infection.

## Introduction

The cytoplasmic tripartite motif protein 5α (TRIM5α) has been shown to restrict the replication of a broad range of retroviruses in a species-specific manner [1]–[5]. For example, TRIM5α of rhesus monkeys mediates an early, post-entry block of HIV-1 replication but only a modest block of SIV replication *in vitro* [4], whereas human TRIM5α restricts N-MLV potently but HIV-1 only weakly [1]–[3],[5]. Although primate TRIM5α variants share a similar domain organization, TRIM5α sequences are highly polymorphic in different species; this interspecies sequence variation of TRIM5α is associated with differences in the viral specificity of TRIM5α-mediated restriction [3],[5],[6],[7]. Multiple *TRIM5* alleles have been recently identified in Old World primates, including rhesus monkeys and sooty mangabeys [8],[9]. While a few of these polymorphic forms of TRIM5α have an effect on the susceptibility of transduced cells to *in vitro* retrovirus infection (HIV-1 and N-MLV), no significant association has been observed between any of these *TRIM5* alleles and either the control of SIV replication *in vivo* or AIDS pathogenesis.

Although they are resistant to infection with HIV-1, rhesus monkeys support the replication of certain strains of SIV and develop an AIDS-like disease following infection with these isolates. The SIV-infected rhesus monkey has become an invaluable model for studying AIDS pathogenesis and evaluating AIDS vaccine strategies [10]–[12]. Interestingly, high levels of viral replication are achieved following infection with SIV in some rhesus monkeys, whereas other rhesus monkeys appear to be more resistant to infection. Certain MHC class I alleles have been associated with efficient control of SIV replication *in vivo* on the basis of particularly effective SIV-specific cytotoxic T lymphocyte responses [13]. Factors other than adaptive immune responses also have been implicated in the control of SIV replication, since the relative permissivity of a monkey's peripheral blood mononuclear cells (PBMCs) for SIV replication *in vitro* predicts the level of SIV replication that occurs *in vivo* when that monkey is infected with SIV [14]. The present study was initiated to explore the contribution of intraspecies *TRIM5* allelic polymorphisms to the control of SIV replication in Indian-origin rhesus monkeys.

## Results

### Sequencing of rhesus monkey TRIM5α

**Figure 1:**
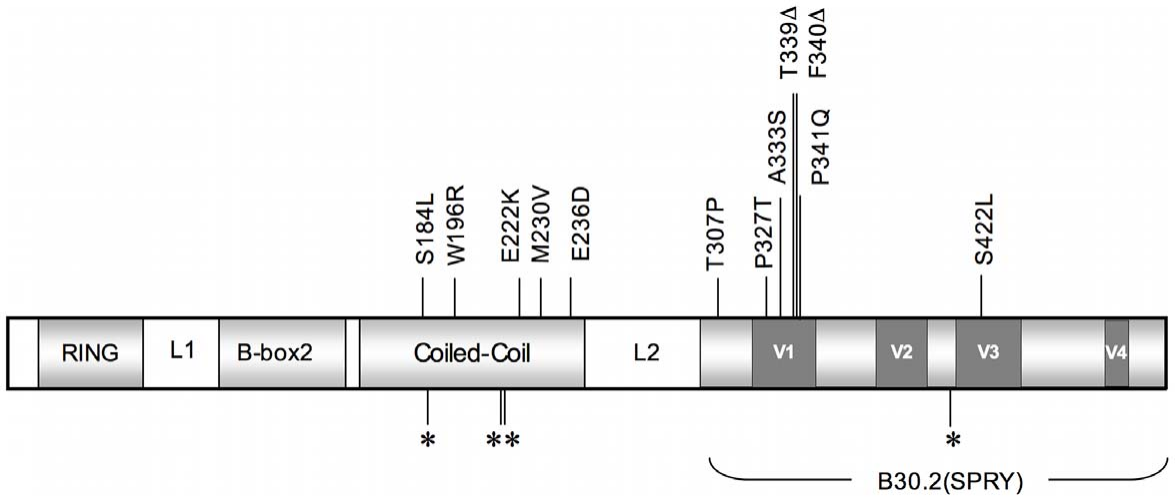
A schematic illustrating TRIM5α and its functional domains. The major domains of TRIM5α are shaded in light grey, and the linker 1 (L1) and linker 2 (L2) regions are designated. The dark grey boxes correspond to variable regions (V1–V4) in the B30.2 (SPRY) domain of the molecule. Locations of coding SNPs are indicated by vertical lines. Nonsynonymous (ns) SNPs with corresponding amino acid changes relative to the major allele are shown above and synonymous SNPs are indicated below by asterisks. These alleles were defined by sequencing full-length cDNA and genomic DNA generated from either B-lymphoblastoid cell lines (B-LCL) or PBMCs of these monkeys.

To characterize the TRIM5α variants of rhesus monkeys, full-length *TRIM5* cDNAs were derived and sequenced from 79 unrelated Indian-origin rhesus monkeys. Six to 15 independent cDNA clones from each monkey were sequenced. Genomic DNAs from these same animals were also sequenced to confirm the identity of these alleles. We identified a 2-amino acid deletion and 14 single nucleotide polymorphisms (SNPs), each of which was present in at least two animals in this cohort of rhesus monkeys (Fig. 1). The 2-amino acid deletion and most of the nonsynonymous SNPs (nsSNPs) clustered in the coiled-coil (CC) and the B30.2(SPRY) domains of the molecule. An allele was identified with a nsSNP in the L2 linker, and 4 *TRIM5* alleles were also characterized that had synonymous polymorphisms clustered in the CC and B30.2(SPRY) regions of the molecule. Based on the distribution of the 10 nsSNPs, the rhesus monkey *TRIM5* coding sequences were grouped into 12 distinct alleles. These alleles and their frequencies in a cohort of 79 monkeys are shown in Table 1, ordered according to increasing numbers of nsSNPs. The predicted amino acid sequence of allele 1 was identical to the published rhesus monkey TRIM5α sequence (AY625001), and the predicted sequence of allele 11 was most divergent from allele 1. TRIMCyp, a molecule in which the TRIM5 RBCC domains are fused to cyclophilin A (CypA) [15]–[18], was cloned from cDNAs prepared from the rhesus monkeys and is designated as allele 12. The coincident expression of two different alleles was observed in many of the monkeys.

**Table 1:** *TRIM5* alleles and their frequencies in Indian-origin rhesus monkeys.

TRIM5α domain	Coiled-coil				L2	B30.2(SPRY)						
nt Change^1^	C551T	T586C	G664A	A688G	A708T	A919C	C979A	G997T	ACGTTT1015–1020------	C1022A	C1265T	Allele Frequency^3^ n/178 (%)
AA Change^2^	S184L	W196R	E222K	M230V	E236D	T307P	P327T	A333S	TF339–340ΔΔ	P341Q	S422L	
Allele 1	S	W	E	M	E	T	P	A	TF	P	S	59/178(33.1)
Allele 2	S	W	E	M	D	T	P	A	TF	P	S	8/178(4.5)
Allele 3	S	W	E	M	E	P	P	A	TF	P	S	8/178(4.5)
Allele 4	S	W	E	M	D	P	P	A	TF	P	S	9/178(5.1)
Allele 5	L	R	K	V	D	T	P	A	TF	P	S	13/178(7.3)
Allele 6	S	R	E	M	E	T	P	S	ΔΔ	Q	L	6/178(3.4)
Allele 7	S	W	E	M	E	P	P	S	ΔΔ	Q	L	19/178(10.7)
Allele 8	S	W	E	M	D	P	P	S	ΔΔ	Q	L	9/178(5.1)
Allele 9	S	W	E	M	D	P	T	S	ΔΔ	Q	L	9/178(5.1)
Allele 10	S	W	K	M	D	P	T	S	ΔΔ	Q	L	7/178(3.9)
Allele 11	L	R	K	V	D	P	T	S	ΔΔ	Q	L	15/178(8.4)
Allele 12^4^	TRIMCyp											16/178(9.0)

1 DNA sequences were aligned with the published sequence of *TRIM5* of the rhesus monkey fibroblast cell line (AY625001). Nucleotide (nt) changes are shown for each allele. Deletions are indicated by dashes.

2 Predicted amino acid (AA) substitutions for nonsynonymous coding SNPs. The amino acid encoded by the predominant allele precedes the residue number, which is followed by the amino acid encoded by the minor allele. Deletions are indicated by Δ.

3 Frequency of *TRIM5* alleles in 79 evaluated Indian-origin rhesus monkeys.

4 TRIMCyp, a molecule in which the TRIM5 RBCC domain is fused to CypA [15]–[18], was cloned and sequenced from cDNA prepared from rhesus monkey lymphocytes.

### TRIM5α polymorphisms are associated with susceptibility of B-LCLs to SIV infection

**Figure 2:**
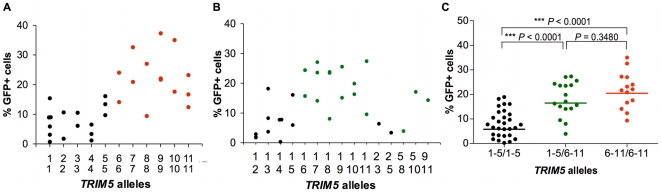
Effect of TRIM5α variants on susceptibility of B-LCLs to SIVmac239 infection. The *TRIM5* alleles expressed by selected rhesus monkey B-LCLs were first characterized by cDNA sequencing. These B-LCLs were then evaluated for their relative susceptibility to SIVmac239 replication by incubation for 48 h with a VSV-G-pseudotyped SIVmac239-GFP construct. The percentage of cells expressing GFP was assessed by flow cytometric analysis in B-LCLs homozygous (A) or heterozygous (B) for *TRIM5* alleles 1–11. The B-LCLs were then divided into three groups: “1–5/1–5” expressing only *TRIM5* alleles 1–5; “1–5/6–11” expressing a *TRIM5* allele from the 1–5 groups and another from the 6–11 groups, “6–11/6–11” expressing only *TRIM5* alleles 6–11 (C). The sequences of amino acid residues 328–479 of the TRIM5α B30.2 (SPRY) domain encoded by alleles 1–5 are identical to each other and to those previously described as rhesus monkey TRIM5α (AY625001). In contrast, a 2-amino acid deletion (339–340) and 3 nsSNPs (A333S, P341Q and S422L) are present in the B30.2 (SPRY) domain of TRIM5α variants encoded by alleles 6–11. The comparison of the values from the 3 groups of B-LCL was analyzed using a non-parametric one-way ANOVA Kruskal-Wallis test with a Dunn's multiple comparison test.

We then sought to assess the impact of this rhesus monkey *TRIM5* allelic diversity on the susceptibility of monkey cells to SIV infection. Since TRIM5α restricts the replication of retroviruses by interfering with a post-entry step in their life cycle, we chose first to evaluate the effect of specific *TRIM5* allelic products by assessing the *in vitro* susceptibility of rhesus monkey B-lymphoblastoid cell lines (B-LCLs) expressing defined *TRIM5* alleles to infection with a vesicular stomatitis virus G (VSV-G)-pseudotyped SIVmac239-GFP construct. The relative *in vitro* susceptibility to SIVmac239 infection of rhesus monkey B-LCLs expressing homozygous or heterozygous *TRIM5* alleles 1–11 is shown in Fig. 2A and B. A significant variation in susceptibility to SIVmac239 infection was observed among these genetically diverse B-LCLs (*P* = 0.0164). We then used a Dunn's post test to compare the relative permissiveness for infection of each group of B-LCLs to that of the B-LCLs that expressed only *TRIM5* allele 1 (Table 2). Rhesus monkey B-LCLs homozygous for *TRIM5* alleles 7, 9 and 10 were significantly more permissive for SIVmac239 infection. The TRIM5α proteins encoded by these alleles share identical B30.2(SPRY) domain residues 328–497. These amino acid residues are also shared by TRIM5α products of alleles 6, 8 and 11. Our inability to demonstrate statistically significant increased permissiveness of alleles 6, 8, and 11 for SIVmac239 infection may be a consequence of the small number of B-LCL populations for evaluation. In fact, when these B-LCLs were assigned to 3 groups on the basis of homozygosity or heterozygosity for expression of alleles 1–5 or 6–11, a statistically significant difference in the permissivity of the cells for SIVmac239 infection was observed (Fig. 2C).

**Table 2:** Comparison of the % GFP values from rhesus monkey B-LCLs expressing various combinations of *TRIM5* alleles with a control group of B-LCLs expressing only the *TRIM5* allele 1.

*TRIM5* alleles		n	Difference in rank sum	*P* value	Significant
Homozygous					
2	2	2	0.963	1.000	No
3	3	2	−1.137	1.000	No
4	4	3	3.467	1.000	No
5	5	3	−5.903	0.986	No
6	6	2	−11.887	0.491	No
7	7	2	−19.587	0.025	**Yes**
8	8	2	−11.042	0.604	No
9	9	3	−22.537	0.006	**Yes**
10	10	2	−17.603	0.018	**Yes**
11	11	3	−10.270	0.494	No
Heterozygous					
1	2	2	4.983	0.998	No
1	3	3	−2.890	1.000	No
1	4	3	1.907	1.000	No
1	5	2	−3.852	1.000	No
1	6	2	−12.887	0.368	No
1	7	3	−14.437	0.092	No
1	8	3	−11.277	0.353	No
1	9	2	−13.187	0.335	No
1	10	2	−10.932	0.619	No
1	11	2	−11.307	0.568	No
2	3	1	0.773	1.000	No
2	5	1	3.763	1.000	No
5	10	1	3.243	1.000	No
5	8	1	−9.937	0.964	No
9	11	1	−7.137	0.999	No

Dunn's multiple comparison test was applied to assess the significance of the difference in GFP expression between each of the groups of B-LCLs and the *TRIM5* allele 1 homozygous B-LCLs.

The observation above suggested that the permissiveness of rhesus monkey B-LCLs for SIVmac239 replication was associated with amino acid changes clustered in the B30.2(SPRY) domain of TRIM5α. The TRIM5α proteins encoded by the more restricted alleles 1–5 are identical in amino acid residues 328–497 in the B30.2(SPRY) domain. These sequences are also identical to that previously described as rhesus monkey TRIM5α AY625001. Interestingly, alleles 6–11 are all identical in residues 328–497 of the B30.2(SPRY) domain, but differ from alleles 1–5 because of a 2-amino acid deletion (339–340) and 3 nsSNPs (A333S, P341Q and S422L). The results suggest that amino acid changes in the region including residues 328–497 of the TRIM5α B30.2(SPRY) domain determine the efficiency of TRIM5α-mediated restriction for SIVmac239 replication in B-LCLs.

### Effects of TRIM5α polymorphisms on retrovirus infections

**Figure 3:**
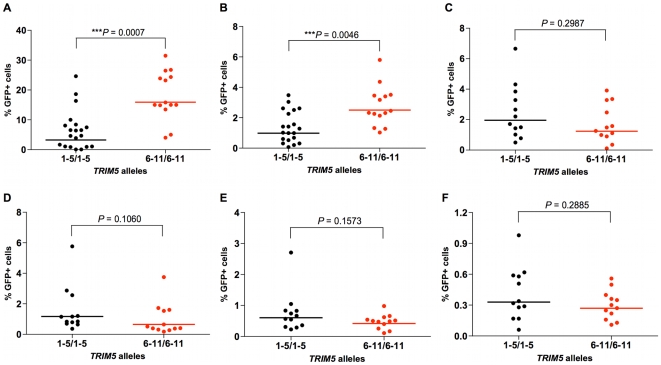
Effects of TRIM5α variants on susceptibility of rhesus monkey B-LCLs to VSV-G-pseudotyped retrovirus constructs. Rhesus monkey B-LCLs expressing selected *TRIM5* alleles were divided into two groups, one expressing only *TRIM5* alleles 1–5, and the other expressing only *TRIM5* alleles 6–11. They were then evaluated for their relative susceptibility to retrovirus infection by incubation for 48 h with VSV-G-pseudotyped recombinant SIVsmE543-GFP (A), HIV-1-GFP (B), EIAV-GFP (C), FIV-GFP (D), N-MLV-GFP (E) or B-MLV-GFP (F) and assessed for % of cells expressing GFP by flow cytometric analysis. The comparison of the values from the 2 groups of B-LCL was analyzed using the Mann-Whitney test.

A number of other complementing observations were made in studying the permissiveness of rhesus monkey B-LCLs assigned to 2 groups on the basis of homozygosity for expression of alleles 1–5 or 6–11 to retrovirus infection. We found that B-LCLs expressing only *TRIM5* alleles 1–5 efficiently restricted SIVsmE543 and HIV-1 infection, whereas B-LCLs expressing only TRIM5α molecule from the group of alleles 6–11 were more permissive for infection by these virus constructs (Fig. 3A, B). The overall infectivity of HIV-1 was substantially lower than that of SIVmac239 in the rhesus monkey B-LCLs. This likely reflects the fact that, even in B-LCLs bearing alleles 6–11, rhesus monkey TRIM5α provides a significant barrier to HIV-1 infection. In contrast to this finding, no significant differences in susceptibility to infection by other retroviruses such as equine infectious anemia virus (EIAV), feline immunodeficiency virus (FIV), N-tropic murine leukemia virus (N-MLV) and B-tropic murine leukemia virus (B-MLV) were found between B-LCLs expressing one or the other groups of *TRIM5* alleles (Fig. 3C–F). These results suggest the rhesus monkey TRIM5α polymorphisms specifically influence restriction potency for a subset of retroviruses that includes the primate immunodeficiency viruses (PIVs).

### TRIM5 allelic variants differ in their ability to restrict VSV-G-pseudotyped SIVmac239-GFP infection of feline fibroblasts

**Figure 4:**
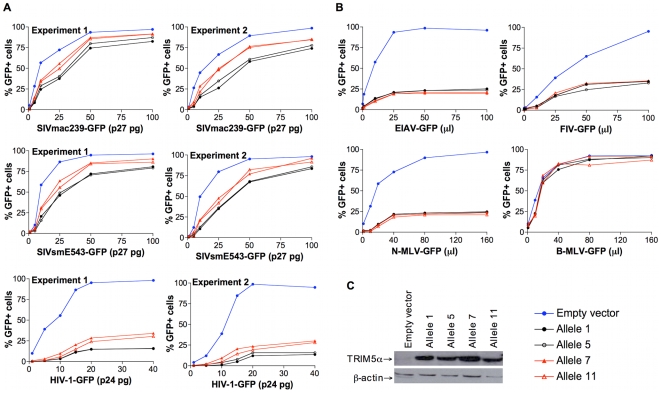
Antiretroviral activity of TRIM5α variants expressed in feline renal fibroblast (CRFK) cells. Feline renal fibroblast (CRFK) cells stably expressing TRIM5α variants encoded by allele 1, 5, 7 or 11, or control cells transduced with the empty pLPCX vector were incubated with a VSV-G-pseudotyped recombinant virus construct for 48 h and then subjected to flow cytometric analysis. (A) To assess the effects of TRIM5α variants on the infection of primate immunodeficiency viruses (PIVs), SIVmac239-GFP, SIVsmE543-GFP, and HIV-1-GFP were tested. The results of a repeat experiment are shown. Six dilutions of the stock of SIVmac239-GFP, SIVsmE543-GFP, and HIV-1-GFP were used to infect the transduced cell lines. (B) CRFK cells expressing different TRIM5α variants or control cells transduced with the empty pLPCX vector were incubated with EIAV-GFP, FIV-GFP, N-MLV-GFP or B-MLV-GFP. Infected GFP-positive cells were assessed by flow cytometric analysis. Represent data from repeated experiments are shown. Six dilutions of EIAV-GFP, FIV-GFP, N-MLV-GFP, and B-MLV-GFP were used to achieve 100% transduction of the control cells with the empty pLPCX vector. (C) The expression of TRIM5α protein by the transduced cells was determined by Western blotting using an anti-HA antibody. Levels of β-actin were also assessed as a control for the loading of total protein.

The contribution of specific rhesus monkey *TRIM5* alleles to the inhibition of SIVmac infection was also assessed in *TRIM5*-transduced cell lines. Feline renal fibroblast (CRFK) cells were stably transduced to express TRIM5α variants encoded by alleles 1, 5, 7 or 11. To produce recombinant viruses, we co-transfected 293T cells with the pLPCX vectors expressing each TRIM5α variant with pVpack-GP and pVPack-VSV-G packaging plasmids. CRFK cells were transduced using the resulting viruses and then selected in G418. These transduced cells were infected with a VSV-G-pseudotyped SIVmac239-GFP construct and subjected to flow cytometric analysis. Consistent with the studies of the B-LCLs, we found that cells transduced with allele 1 or 5 supported a lower level of infection by SIVmac239-GFP, whereas cells transduced with allele 7 or 11 supported a higher level of SIVmac239 infection (Fig. 4A). We also assessed the infectivity of VSV-G-pseudotyped GFP-expressing SIVsmE543, HIV-1, EIAV, FIV, N-MLV and B-MLV viruses these same transduced cells. The restriction of SIVsmE543 and HIV-1 infection was more efficient in cells transduced with allele 1 or 5 than in cells transduced with allele 7 or 11 (Fig. 4A). While transduction of cells with the *TRIM5* alleles inhibited infection by EIAV, FIV and N-MLV, all evaluated *TRIM5* alleles inhibited the infection with comparable efficiency (Fig. 4B). Expression of these alleles did not restrict B-MLV infection (Fig. 4B). We verified that the HA epitope tag at the C terminus of the TRIM5α protein did not affect the ability of transduced cells to restrict SIVmac239 infection (data not shown). We also confirmed that downregulation of TRIM5α expression by siRNA specifically rescued the infectivity of the restricted SIVmac239 (data not shown). Since the level of TRIM5α protein expression in each of the transduced cells was comparable as determined by Western blotting, differences in TRIM5α protein expression level could not account for the observed differences in the ability of the cells to restrict SIVmac239 infection. The TRIM5α proteins encoded by allele 1 and 7, and by alleles 5 and 11, differ only in the sequences of the B30.2(SPRY) domain (Table 1). Therefore, as in the studies of B-LCLs, the permissiveness for SIVmac replication in the transduced cells was associated with amino acid changes clustered in the B30.2(SPRY) domain of TRIM5α.

### Effects of TRIM5α polymorphisms on SIVmac239 capsid binding

We then explored the mechanism accounting for the differences in efficiency of restriction of SIVmac239 infection mediated by these different *TRIM5* alleles. A direct interaction of TRIM5α with the retroviral capsid is required for restriction of retrovirus replication [19]–[21]. The recognition of the capsid by TRIM5α is mediated by the B30.2(SPRY) domain. Because the phenotypic differences among rhesus monkey TRIM5α variants grouped according to the sequence of the B30.2(SPRY) domain, we hypothesized that the two groups of TRIM5α variants might exhibit differences in the ability to bind SIVmac capsids. Two TRIM5α proteins encoded by alleles 1 and 7 were studied for capsid-binding ability. *TRIM5* allele 1 represents the major allele and encodes a protein identical in sequence to that of the previously reported rhesus monkey TRIM5α [4]. The TRIM5α protein encoded by allele 7 differs from that encoded by allele 1 only in the sequence of the B30.2(SPRY) domain (Table 1). Therefore, TRIM5α variants 1 and 7 were chosen as representatives of the two groups of TRIM5α B30.2(SPRY) domain variants and tested for their ability to bind SIVmac capsid.

**Figure 5:**
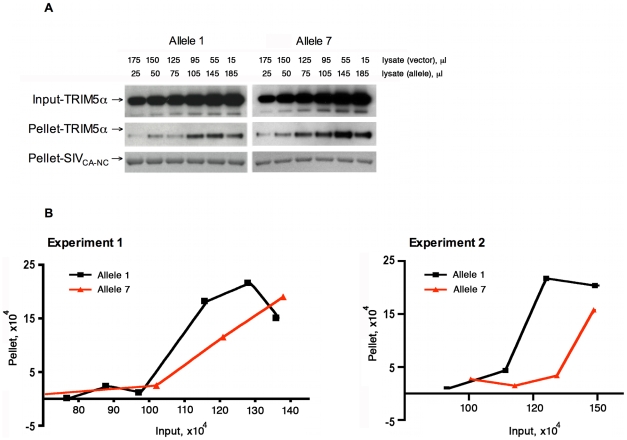
Effect of TRIM5α variation on SIVmac239 capsid binding. Serial dilutions of 293T cell lysates (input) containing TRIM5α-HA proteins encoded by allele 1 or allele 7 were incubated with equal amounts of SIVmac239 CA-NC complexes assembled *in vitro*. After incubation, the mixtures were pelleted through a sucrose cushion. The amounts of TRIM5α in both input and pellet were subjected to Western blotting and densitometry. The results of two separate experiments are shown. The Western blot (A) of experiment 1 was quantitatively analyzed by a densitometer with the results shown in (B). The last two samples of the allele 7 variant were beyond the linear range of the densitometry and, therefore, were not included in the final analysis in (B). Both pellet and input amounts are shown in arbitrary densitometric units.

For this purpose, we adapted an *in vitro* capsid-binding assay, which has been used to study TRIM5α binding to the HIV-1 capsid [22]. In our assay, SIVmac239 capsid-nucleocapsid (CA-NC) complexes were generated *in vitro*, and then incubated with serial dilutions of 293T cell lysates containing TRIM5α allelic variants 1 and 7. After incubation, TRIM5α protein bound to CA-NC complexes was separated from unbound TRIM5α by pelleting through a sucrose cushion. The amount of TRIM5α in both input and pellet was analyzed by Western blotting and densitometry. As expected from previous experience with the HIV-1 capsid-nucleocapsid binding assay [23], both TRIM5α allelic variants bound the SIVmac239 CA-NC complexes in a dose-dependent manner with a sigmoidal pattern. Importantly, however, binding of the variant encoded by *TRIM5* allele 7 was less efficient than that of the TRIM5α allele 1 variant (Fig. 5A, B). This finding is consistent with the possibility that differences in the efficiency of binding the SIVmac239 capsid contribute to the observed differences in SIVmac239 restriction potency, with the less efficiently binding allelic variant 7 mediating the less potent restriction of viral replication.

### TRIM5α polymorphisms are associated with plasma viral RNA levels and clinical sequelae of SIVmac251 infection

To determine the ramifications of these *in vitro* findings for the control of viral replication *in vivo*, a study was done to determine whether the expression of particular *TRIM5* alleles by Indian-origin rhesus monkeys was associated with the *in vivo* control of SIVmac251 replication. We tested the specific hypothesis that variation in residues 328–497 of the TRIM5α B30.2(SPRY) domain influences the course of SIVmac251 infection *in vivo*. The monkeys were divided into 3 groups: in one group, only *TRIM5* alleles 1–5 were expressed; in the second group, one copy of alleles 1–5 and one copy of alleles 6–11 were expressed; and in the third group, only alleles 6–11 were expressed. Monkeys were selected for this study that did not express the MHC class I alleles Mamu-A*01, -B*08, or -B*17, as their expression is associated with particularly efficient control of SIV replication [24]–[26]. Eliminating these monkeys from the evaluated animals eliminated an already defined source of variation in virus control.

**Figure 6:**
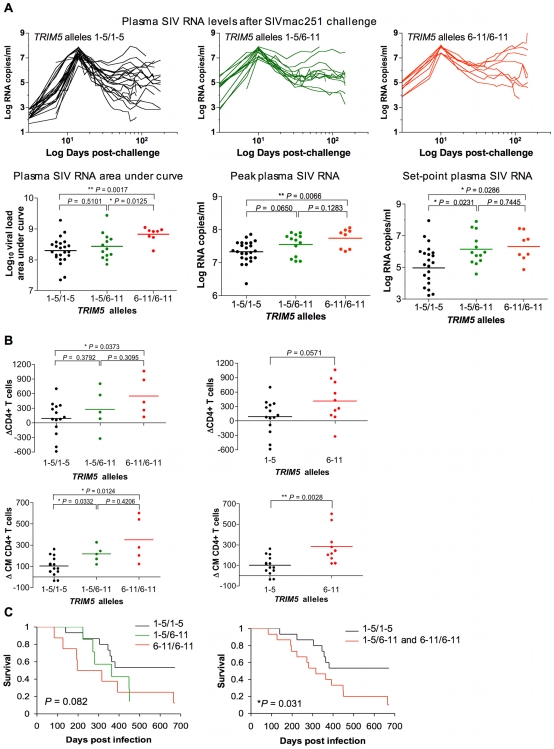
Association of the expression of groups of *TRIM5* alleles by rhesus monkeys with viral replication following SIVmac251 infection. (A) Plasma virus RNA levels were assessed in a cohort of Indian-origin rhesus monkeys between days 1 and 178 after challenge. Area-under-the curve calculations for the plasma SIV RNA levels were assessed in these monkeys. The plasma SIV RNA levels were also assessed on days 14 and 70 following challenge, representing peak and set-point viral load, respectively. (B) The loss of total CD4+ T cells and central memory CD4+ T cells was monitored in the same cohorts of rhesus monkeys following SIVmac251 infection. Data are displayed two ways: dividing the monkeys into 3 cohorts as described in the legend to Fig. 2 or dividing the monkeys into 2 cohorts, one of animlas expressing only alleles 1–5 and the other of animals expressing at least 1 allele of the groups 6–11. (C) The survival of monkeys following SIVmac251 infection is shown in Kaplan-Meier (KM) curves. The *P*-value corresponds to the log-rank test of equality of the survival curves. The monkeys are divided into either 3 or 2 groups as described above. The comparison of the values from the groups of monkeys was analyzed using a non-parametric one-way ANOVA Kruskal-Wallis test with Dunn's multiple comparison test or the Mann-Whitney test.

Plasma SIV RNA levels were assessed in this cohort of rhesus monkeys through day 178 after SIVmac251 challenge. We measured viral replication for each monkey by doing an area-under-the curve calculation for the plasma SIV RNA levels between days 1 and 70 following infection. A 0.1 log median difference in these values was observed between the allele 1–5 homozygous and the allele 1–5/allele 6–11 heterozygous monkeys, and a 0.6 log median difference in these values was observed between the allele 1–5 homozygous and the allele 6–11 homozygous monkeys (Fig. 6A). Plasma virus RNA levels were also assessed in this cohort of rhesus monkeys on days 14 (peak) and 70 (set-point) following the intravenous inoculation of SIVmac251. Consistent with the decreased *in vitro* antiviral activity of the products of *TRIM5* alleles 6–11, the expression of *TRIM5* alleles 6–11 by the rhesus monkeys was associated with higher levels of SIVmac251 replication *in vivo*. A 0.4 log median difference in plasma virus RNA levels at the time of peak viral replication was observed between the allele 1–5 homozygous and the allele 1–5/allele 6–11 heterozygous monkeys, and a 0.6 log median difference in these values was observed between the allele 1–5 homozygous and the allele 6–11 homozygous monkeys (Fig. 6A). A log median difference in set-point virus RNA levels was 0.7 between the allele 1–5 homozygous and the allele 1–5/allele 6–11 heterozygous monkeys, and 1.3 between the allele 1–5 homozygous and the allele 6–11 homozygous monkeys (Fig. 6A).

We then assessed the clinical consequences of infection in these monkeys. First, we evaluated the loss of peripheral blood total CD4+ T cells and CM CD4+ T cells in the monkeys following SIVmac251 inoculation. We sought to determine whether the expression of particular *TRIM5* alleles was associated with particular immune sequelae of infection. We displayed the data in two ways: dividing the monkeys into 3 cohorts as described above and dividing the monkeys into 2 cohorts, one group of animals expressing only alleles 1–5 and the other expressing at least 1 allele of the group 6–11. The expression of *TRIM5* alleles 6–11 by the rhesus monkeys was associated with a rapid loss of both total CD4+ T cells and CM CD4+ T cells (Fig. 6B). Then we assessed the effect of the expression of *TRIM5* alleles 6–11 on the survival of the monkeys following infection (log-rank test of equality, Kaplan-Meyer survival curves). The monkeys expressing only alleles 1–5 maintained a statistically significant survival advantage over the monkeys expressing at least 1 allele of the group 6–11 (Fig. 6C). Therefore, the expression of *TRIM5* alleles 6–11 was associated with both less efficient control of SIVmac replication *in vivo* and an increased rate of disease progression.

## Discussion

We demonstrated *TRIM5* allele-determined relative resistance to SIV infection *in vitro* using both B-LCL and transduced fibroblast target cells. However, the variable resistance or permissiveness for infection was more readily apparent in the B-LCL than in the fibroblasts. This difference between target cell populations may reflect differences in *TRIM5* expression under normal physiologic control in B-LCLs and in transduced CRFK cells. Thus, overexpression in CRFK cells may partially mask the relative inefficiency of some of the *TRIM5* alleles to restrict SIV and HIV replication *in vitro*.

A number of genes have previously been implicated in the control of HIV-1 replication. These include selected MHC class I alleles and loci, including HLA B*5701, HLA B*27 [27] and HLA C [28], as well as HLA Bw4-8OI in association with KIR3DS1 [29]. Expression of an allelic form of the HIV-1 co-receptor, the delta 32 form of CCR5, is also associated with HIV-1 containment [30]–[32]. These genes and gene loci are thought to contribute to HIV-1 control through classical immune effector mechanisms or viral entry.

The present study shows that naturally occurring TRIM5α B30.2(SPRY) polymorphisms affect immunodeficiency virus infections. These observations demonstrate the importance of SIV/B30.2(SPRY) interactions *in vivo*. The finding that *TRIM5* allelic products are associated with both the permissiveness of cells to HIV-1/SIV infection *in vitro* and clinical sequelae of SIV infection *in vivo* expands the known genetic determinants of HIV-1/SIV susceptibility and implicates a novel intracellular mechanism in that virus control.

Our data suggest that the co-expression of restrictive and permissive TRIM5α variants results in a less effective antiretroviral state than the expression of two restrictive TRIM5α variants. Differences in SIVmac restriction potency and capsid binding map to the B30.2(SPRY) domain of the rhesus monkey TRIM5α variants. As the functional TRIM5α moiety is thought to an oligomer [33]–[36], co-expression of TRIM5α variants with different capsid-binding affinities would be expected to result in heterodimers with decreased avidity for the capsid. Dominant-negative effects of less functional TRIM5α variants on more potently restricting TRIM5α variants have been observed [4], [37]–[39].

A number of groups have evaluated the contribution of common TRIM5α polymorphisms to AIDS susceptibility in HIV-1-infected humans [40]–[44]. These various studies have defined associations of nsSNPs in the RING, B-box 2, Coiled-coil domains and Linker 2 region of the molecule with very modest effects on HIV-1 susceptibility and AIDS clinical progression [40]–[43]. The single SPRY domain ns SNP in human TRIM5α, H419Y, was also shown to have only a modest effect on these clinical endpoints [44]. The absence of evidence for a robust association between a SNP in TRIM5α and a substantial HIV-1-associated clinical endpoint in humans is likely a consequence of the fact that the common variant forms of this molecule in humans do not have severely impaired interactions with retroviruses. The human *TRIM5* SNPs are for the most part clustered in the B-box 2 and Coiled-coil domains of the molecule, regions of TRIM5α that are not thought to contact the retrovirus capsid. In contrast, as we have shown in the present study, variants of TRIM5α in the rhesus monkey that cluster in the B30.2 (SPRY) domain of the molecule have a significant impact on the interaction of TRIM5α with the capsid of SIV and, as a result, exert a significant effect on SIV replication *in vitro and in vivo*.

The findings in the present study indicate that TRIM5α has a function beyond restricting cross-species transmission of retroviruses. We show that TRIM5α variants with an impaired ability to interact with the retrovirus capsid restrict pathogenic retroviruses in a susceptible host species less efficiently both *in vitro* and *in vivo*. Therefore, TRIM5α proteins can exert antiretroviral effects ranging from modifications of viral load to complete suppression of infection.

## Methods

### Cells

HEK293T and feline renal fibroblasts (CRFK) were obtained from American Type Culture Collection and grown in RPMI/10% FBS.

### Recombinant viruses

Recombinant SIVmac239-GFP virus was produced by cotransfection of HEK293T cells with pSIVmac239Δenv-GFP, pVSV-G and pRev using the Lipofectamine 2000 (Invitrogen). HIV-1-GFP, SIVsmE543-GFP, EIAV-GFP, FIV-GFP, B- and N-MLV-GFP viruses were prepared as previously described [7]. At 48h after transfection, cell-free supernatant was collected. The titer of virus in the supernatant was determined by infection of HEK293T cells and assessment for % of cells expressing GFP by flow cytometric analysis. An SIV p27 antigen ELISA was performed to measure the p27 *gag* gene product of SIV.

### Establishment of B Lymphoblastoid Cell Lines (B-LCLs)

B-LCLs were generated from the same cohort of 28 uninfected rhesus monkeys and 32 rhesus monkeys previously infected with SIVmac251 as described [45]. Briefly, B cells were transformed by incubating peripheral blood mononuclear cells isolated from rhesus monkeys with *Herpesvirus papio*. B-LCLs were then propagated in RPMI 1640 supplemented with 20% FBS and penicillin-streptomycin.

### Sequencing of TRIM5α cDNA

Total RNA was isolated from *H. papio*-immortalized rhesus monkey B-LCLs and PBMCs by using the RNeasy Mini kit (Qiagen). Untagged, full-length TRIM5α cDNA clones were generated by RT-PCR with primers TRIM5F1 (5′-CAGACGAATTCCACCATGGCTTCTGGAATCCTG-3′) and TRIM5R1 (5′-GGACGTTCGAAATAGAAAGAAGGGAGACAGC-3′) by using the SuperScript One-Step kit (Invitrogen). PCR products were cloned directly into the TOPO Blunt vector (Invitrogen). Six to 15 independent cDNA clones from individual rhesus monkeys were subjected to automated sequence analysis (GENEWIZ). Genomic DNA was isolated from lymphocytes from the same cohorts of rhesus monkeys by using QIAamp DNA kit (Qiagen), and sequenced. Primer selection was facilitated by the use of the computer program Beacon 3. Primer sequences used to amplify and sequence *TRIM5* exons or *TRIM5* cDNAs are shown in Table S1. To generate TRIM5α C-terminally tagged with hemagglutinin (HA), genes were amplified with primers TRIM5F1 and TRIM5HA-R (5′-CCACCGGTGGCTCAAGCGTAGTCTGGGACGTCGTATGGGTAGCCGCCAGAGCTTGGTGAGCACAGAG-3′).

### Rhesus monkey B-LCL infection

B-LCLs were selected for study that had previously defined *TRIM5* alleles. A VSV-G pseudotyped recombinant virus constructs were used for single-round infection. 4×10^4^ cells per well were seeded into 96-well plates and cells were infected with serial dilutions of virus by spinoculation for 2 h at 1,900×g. At 48 h after infection, these B-LCLs were evaluated for their relative susceptibility to retrovirus replication by assessing for the % of cells expressing GFP as determined by flow cytometric analysis.

### Restriction in CRFK cells expressing TRIM5 alleles

Feline renal fibroblast (CRFK) cells stably expressing TRIM5α variants encoded by allele 1, 5, 7 or 11 were generated as described by Stremlau et al. [4]. Briefly, 293T cells were co-transfected with the pLPCX vectors containing each TRIM5α cDNA with pVpack-GP and pVPack-VSV-G packaging plasmids (Stratagene) to produce recombinant viruses. CRFK cells were then transduced using the resulting viruses and selected in G418 (0.5mg/ml, Sigma-aldrich). For infection, CRFK cells expressing TRIM5α variants encoded by allele 1, 5, 7 or 11, or control cells transduced with the empty pLPCX vector were harvested and subsequently seeded into 48-well plates (1.2×10^4^ cells per well). Cells were incubated with a VSV-G-pseudotyped recombinant virus construct for 48 h and then subjected to flow cytometric analysis.

### Immunoblotting

Feline renal fibroblast (CRFK) cells were transduced as described above and harvested. Cells were then lysed in NP-40 lysis buffer (Boston BioProducts). Total protein was measured by using a BCA assay kit (Pierce) and equal amounts (20 µg) were separated by SDS/PAGE. HA-tagged TRIM5α was detected with either monoclonal anti-HA antibody (Roche) and HRP-conjugated either anti-rat or anti-rabbit IgG secondary antibody, respectively (Sigma-Aldrich). Levels of β-actin were also assessed as a control for the loading of total protein by using anti-β-actin antibody (Sigma-Aldrich).

### Capsid-binding assay

The SIVmac239 CA-NC fusion protein was expressed in *Escherichia coli* and purified with minor modifications from the previously described purification method used for the HIV-1 CA-NC protein [22]. The purified SIVmac CA-NC protein was mixed with (TG)^50^ DNA oligonucleotides in 50 mM Tris-HCl (pH 7.0) and 500 mM NaCl solution and incubated at 37°C. The resulting SIVmac CA-NC complexes were negatively stained and examined under the electron microscope, confirming that the size and shape of the CA-NC complexes are very similar to those HIV-1 CA-NC complexes.

Approximately 1×10^7^ 293T cells were transiently transfected to express the appropriate *TRIM5* allele or with a control vector, and then harvested at 24 h post-transfection. The cells were lysed in 1 ml of hypotonic lysis buffer (10 mM Tris-HCl, pH 7.0/10 mM KCl). After brief centrifugation at 4°C to remove cell debris, the supernatant was spun at 110, 000×g for 1 h at 4°C in a Beckman ultracentrifuge. After the pre-cleaning spin, different amounts of the supernatants containing the TRIM5α variants were aliquoted in separate tubes and were complemented with the supernatants from the vector-transformed cells to achieve a final volume of 200 µl. To these mixtures, the same amount of SIVmac CA-NC complexes were added and the final concentration of NaCl was adjusted to 200 mM before incubation at 30°C for 1 h. After incubation, 20 µl of the mixtures were saved for further analysis as an input and the rest of mixtures were layered onto 60% sucrose cushion (prepared in 1× PBS) and centrifuged at 110,000×g for 1 h at 4°C in a Beckman ultracentrifuge. The pellet was resuspended in 1× SDS sample buffer and subjected to SDS/PAGE and Western blotting.

### Monoclonal antibody (MAb) staining of cell surface molecules and CD4+ T-lymphocyte counts

Peripheral blood mononuclear cells (PBMCs) were stained with MAbs specific for cell surface molecules, including CD3, CD4, CD28, and CD95, and then subjected to flow cytometric analysis. Peripheral blood CD4+ T lymphocyte and central memory CD4+ T lymphocyte counts were calculated by multiplying the total lymphocytes count by the percentage of CD3+CD4+ T cells, and CD95+CD28+ T cells, respectively, determined by MAb staining and flow cytometric analysis [46].

### *In vivo* infection

#### Animals

Indian-origin rhesus monkeys used in the analysis were maintained according to the guidelines of the *NIH Guide to the Care and Use of Laboratory Animals* and the approval of the Institutional Animal Care and Use Committee of Harvard Medical School and the National Institute of Health.

#### Challenge virus

A stock of uncloned SIVmac251 was expanded on human PBMC and titered *in vivo* in rhesus monkeys for use in intravenous challenge studies [47]. All monkeys used in the retrospective analysis were received 1 ml of a 1∶3000 dilution of this stock by intravenous route.

#### Plasma viral RNA assay

Assays were performed using an ultrasensitive branched DNA amplification assay (Bayer Diagnostics).

### Statistical analysis

The two-sided nonparametric Mann-Whitney test was used to determine statistical significance. Levels of *P* <0.05 were considered statistically significant. For multiple comparisons, non-parametric one-way ANOVA Kruskal-Wallis test was used with Dunn's multiple comparison test.

## Supporting Information

Table S1 TRIM5α sequencing primers used in the study
(0.05 MB PDF)
