# Supplementary material for: TRIM5α Modulates Immunodeficiency Virus Control in Rhesus Monkeys
Source: PLoS Pathog. 2010 Jan 22;6(1):e1000738. doi: 10.1371/journal.ppat.1000738 (PMC2809762; doi:10.1371/journal.ppat.1000738)
Supplement: Table S1 — TRIM5α sequencing primers used in the study (0.05 MB PDF) [file ppat.1000738.s001.pdf]

Supplemental Table 1. TRIM5α sequencing primers

A. *TRIM5* cDNA sequencing	
Primers	Sequences
M13f	gtaaaacgacggccagt
M13r	aacagctatgaccatg
TRIM5 F2	gcggaattcgccatggcttctggaatc
TRIM5 R2	gcgatcgatgcctcaagagcttggtgagcacag
TRIM5 F3	ggaagctgacatcagaga
TRIM5 R3	tctacctcccagtaatg
TRIM5 F4	gataagagacaagtgagc
TRIM5 R4	tccttctccaggttttgc

B. *TRIM5* genomic DNA sequencing

a. PCR primers

TRIM5 region	Primer orientation	Primers	Sequences
Exon 2-4	Forward	9507	gaacaagacgaacctcagcagcc
	Reverse	11537	gtgctcaggcttctttcccctttttac
Exon 5-6	Forward	23156	ggaaatctcacatgagtcttctggg
	Reverse	25596	agttaggggatataatgtgactttat
Exon 6-8	Forward	24761	gagaacattttgtttgcctccagg
	Reverse	26286	ttcaggtgtatgagatgcacatgg

b. Sequencing primers

TRIM5 region	Primers	Sequences
Exon 2	9536	ggcaggagcagtggagaagctactatg
Exon 3	10431	ccaagtcatggattctcattgcc
Exon 4	11261	cagcctctgcctggtagactgag
Exon 5	23301	atttcttaattgatgttttctctcttcttcctag
Exon 6	24914	gggacacaaggtcagaggtttgg
Exon 7	25264	ctaccatagggcatacctactctttccc
Exon 8	25616	tatatcccctaactgacctgttaatttttctacag
	25941	caacctaaatatggctactgggttatagg
